# The Effect of Increased Neutrophil Lymphocyte Ratio on Mortality in Patients Operated on Due to Hip Fracture

**DOI:** 10.7759/cureus.6543

**Published:** 2020-01-02

**Authors:** Ahmet Atlas, Erdogan Duran, Başak Pehlivan, Veli F Pehlivan, Mehmet K Erol, Nuray Altay

**Affiliations:** 1 Anesthesiology, Harran University, Sanliurfa, TUR; 2 Anesthesiology and Reanimation, Harran University, Sanlıurfa, TUR; 3 Anesthesiology and Critical Care, Harran University, Sanliurfa, TUR; 4 Anesthesiology and Reanimation, Harran University, Sanliurfa, TUR

**Keywords:** hip fracture, neutrophil lymphocyte ratio (nlr), intensive care, mortality

## Abstract

Introduction

In this study, we aimed to examine the effect of neutrophil-lymphocyte ratio (NLR) on mortality and morbidity in elderly patients over the age of 65 who presented to our clinic and were operated on due to hip fracture.

Methods

The study included patients over the age of 65 who were operated on in our hospital between January 2014 and December 2018 due to hip fracture. Those with multiple fractures and those who were operated on due to cancer-related fracture were excluded. Patients' age, gender, American Society of Anesthesiologists (ASA) score, preoperative waiting time, type of anesthesia, operation duration, amount of erythrocyte suspension used, and duration of intensive care unit (ICU) stay were recorded. The effect of increased preoperative and postoperative 5th day neutrophil-lymphocyte ratios (NLR 1 and NLR 5, respectively) on mortality and morbidity was investigated.

Results

We examined 132 patients operated on due to hip fracture. NLR 5 was higher among patients who were admitted to the ICU (p = 0.007) and among those who died (p = 0.007). Additionally, the rate of increase of NLR 5 was higher among patients who were admitted to the ICU (p = 0.044) and among those died (p = 0.009).

Conclusion

The rate of increase of NLR in the postoperative period can be used as a criterion for predicting mortality in patients who are operated on due to hip fracture.

## Introduction

Physiological changes in organ functions and increases in comorbidities that occur with advancing age are the primary causes of postoperative complications [1]. Comorbid diseases, and cardiovascular diseases in particular, increase the risks of surgery and anesthesia [2]. One important cause of morbidity and mortality in the elderly population is hip fracture. One-year mortality rates after hip fractures have been reported in the range of 8.6% to 36%. Major causes of mortality include myocardial infarction, cardiac failure, pneumonia, and pulmonary embolism. Increased neutrophil-lymphocyte ratio (NLR) in the postoperative period after hip fracture operations has been reported as a risk factor for postoperative mortality and cardiovascular complications [3].

NLR is a convenient and readily available biomarker. Hip fracture, which is common and presents as a surgical emergency in elderly patients, is associated with high morbidity and mortality. NLR is significant for predicting mortality and morbidity in the early period following hip fracture. Forget et al. reported that a postoperative 5th day NLR value >5 following hip fracture surgery was associated with high mortality risk [3]. Temiz and Ersözlü found that postoperative NLR value ≥4.7 increased the risk of mortality in patients with hip fracture. Cook et al. found that an NLR value ≥9.3 on the first postoperative day following colorectal surgery was associated with increased mortality [4].

In our review of the literature, we did not encounter any study which examined the postoperative increase in NLR and its effect on mortality in patients aged over 65 years operated on due to hip fracture. In the present study, we aimed to examine preoperative and postoperative 5th day NLR values and to investigate the association of increased NLR with mortality.

## Materials and methods

After obtaining approval of the Ethics Committee, we retrospectively reviewed 132 cases aged 65 years and above who were operated on due to hip fracture in our hospital between January 2014 and December 2018. Patients’ age, gender, American Society of Anesthesiologists (ASA) score, preoperative waiting time, type of anesthesia, amount of erythrocyte suspension used, and duration of stay in the intensive care unit (ICU) were recorded. Comorbid systemic diseases such as coronary artery disease, cardiac failure, chronic obstructive pulmonary disease, cerebrovascular disease, hypertension, diabetes mellitus and renal failure were classified according to ASA classification. Operations performed within two days of hip fracture were regarded as emergency operations, whereas those performed after two days were regarded as elective operations. The association of the increase in preoperative (NLR 1) and postoperative 5th day (NLR 5) NLR values with mortality was investigated.

Parameters were analyzed using SPSS for Windows version 23.0 (IBM Corp., Armonk, NY). Continuous variables are expressed as mean ± standard deviation or median (minimum-maximum). Count and percentage are used to express categorical variables. The difference between the continuous variables in the two groups was analyzed with Mann-Whitney U test. Repeated measures were compared using Wilcoxon test. Chi-square test was used to analyze categorical parameters. Spearman’s Rho correlation test was used to calculate correlation between two continuous variables. A p-value <0.05 was accepted as statistically significant.

## Results

Mean patient age was 80 ± 8.4 years. Mean age of discharged patients was 79.6 ± 8.2 years, whereas the mean age of deceased patients was 94.7 ± 4.7 years. All of the deceased patients were aged 90 years or above (p = 0.005).

Of all the patients who were operated on for hip fracture, 60.6% (n = 80) were women and 39.4% (n = 52) were men. Of the discharged patients, 59.7% (n = 77) were women and 40.3% (n = 52) were men; all three patients who died were women. No significant association was found between gender and mortality (p = 0.278) (Table 1).

**Table 1:** Demographical data ASA: American Society of Anesthesiologists

Demographical data	n (%)
Gender n (%)	
Female	80 (60.6)
Male	52 (39.4)
ASA n (%)	
II	64 (48.5)
III	62 (47)
IV	6 (4.5)

With regard to preoperative waiting time, 89.4% (n = 118) of all patients were operated on electively, while 10.6% (n = 14) were operated on as emergency cases. Two emergency cases and one elective case (0.8%) died. One of the two emergency cases died in the intensive care unit on the 5th postoperative day and the second patient died on the 7th day in the intensive care unit. The elective patient died in the intensive care unit on the 7th postoperative day. The mortality rate was higher among those who underwent emergency surgery (p = 0.030) (Table 2).

**Table 2:** Operation data ICU: Intensive Care Unit

Operation data	Mean ± SD
Hemoglobin (mg/dl)	12.06 ± 1.96
Number of transfused units	1.7 ± 1.1
Preop waiting time (days)	2.1 ± 1.7
Operation time (hours)	2.2 ± 0.6
Duration of ICU stay	1.5 ± 1.7
Duration of hospital stay	10.3 ± 4.9

As for the type of anesthesia, 69.5% of cases operated on as elective cases received general anesthesia while 30.5% received regional anesthesia. Of those cases operated on as emergency cases, 78.6% received general anesthesia while 21.4% received regional anesthesia. Similar NLR 1 values were found between deceased and discharged patients at the time of admission to hospital. However, deceased patients showed greater increase in NLR 5 values (p = 0.007) (Table 3).

**Table 3:** Change in NLR NLR: Neutrophil-Lymphocyte Ratio

	All patients	Discharged	Deceased	p
	Mean ± SD/Median			
NLR				
Preop (NLR1)	7.6 ± 4.6/6.4	7.6 ± 4.5/6.4	7.5 ± 6.7/9.7	0.861
Postop (NLR5)	11.7 ± 7.4/9.7	11.3 ± 7.02/9.5	28.3 ± 6.4/30.4	0.007
Preop-postop change rate	4.1 ± 7.6/2.3	3.7 ± 7.2/2.06	20.7 ± 9.5/20.4	0.009

The change between preoperative and postoperative NLR was greater in deceased patients when compared to discharged patients (p = 0.009) (Table 3).

NLR 1 was similar between patients who were admitted to the ICU and ward (p = 0.152). Both NLR 5 and the change between preoperative and postoperative NLR values were higher in patients who were admitted to the ICU (p = 0.007 and p = 0.044, respectively).

**Figure 1:**
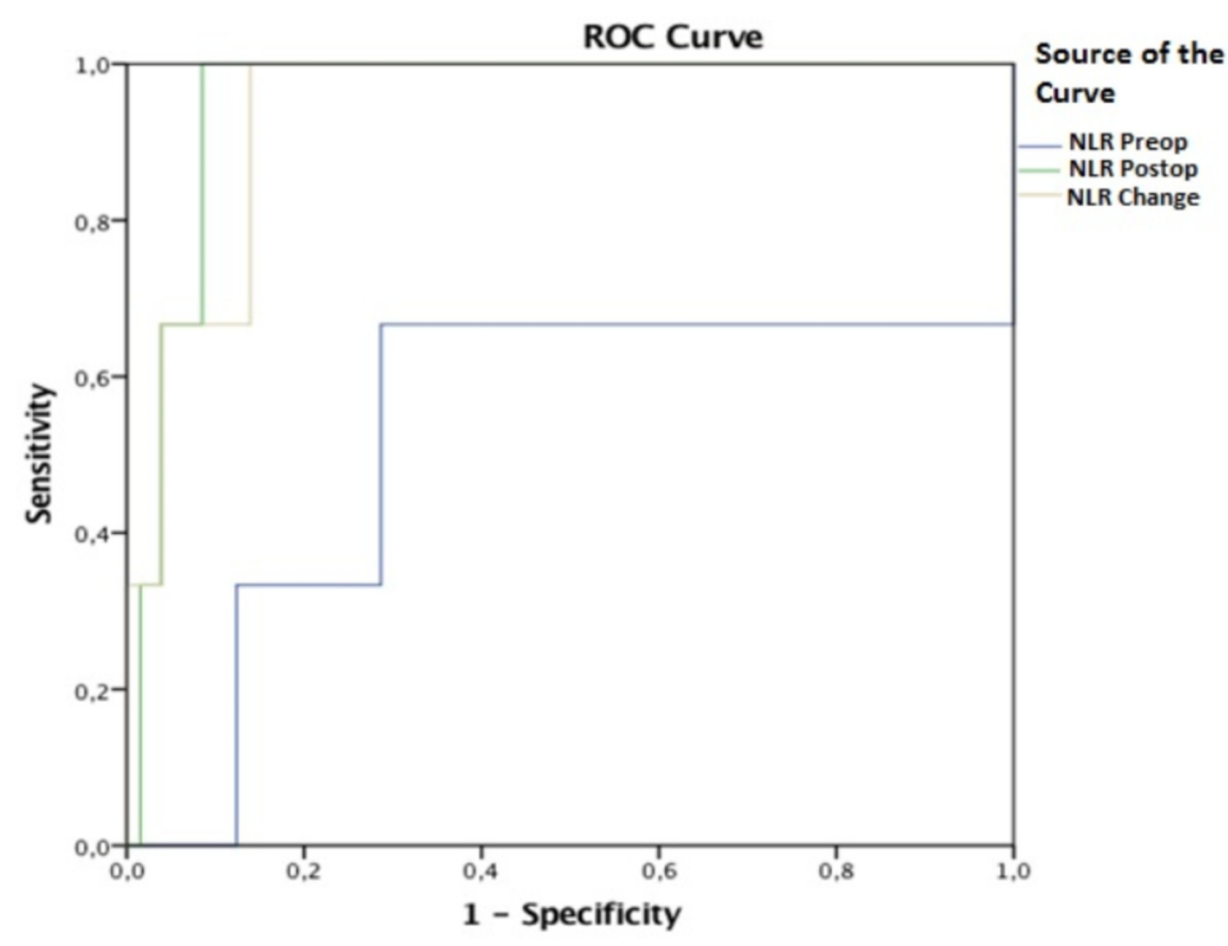
ROC curve Area under curve for preop NLR: AUC = 0.530 (0.097-0.963), (p = 0.861). Best cut-off level for preop NLR: 9.635. Sensitivity and specificity at this level were 66.7% and 69.0%, respectively. Area under curve for postop NLR: AUC = 0.953 (0.908-0.999), (p = 0.007). Best cut-off level for postop NLR: 21.07. Sensitivity and specificity at this level were 100% and 91.5%, respectively. Area under curve for NLR change: AUC = 0.941 (0.867-1.000), (p = 0.009). Best cut-off level for NLR change: 11.25. Sensitivity and specificity at this level were 100% and 85.3%, respectively. ROC: Receiver Operating Characteristic; NLR: Neutrophil to Lymphocyte Ratio.

**Figure 2:**
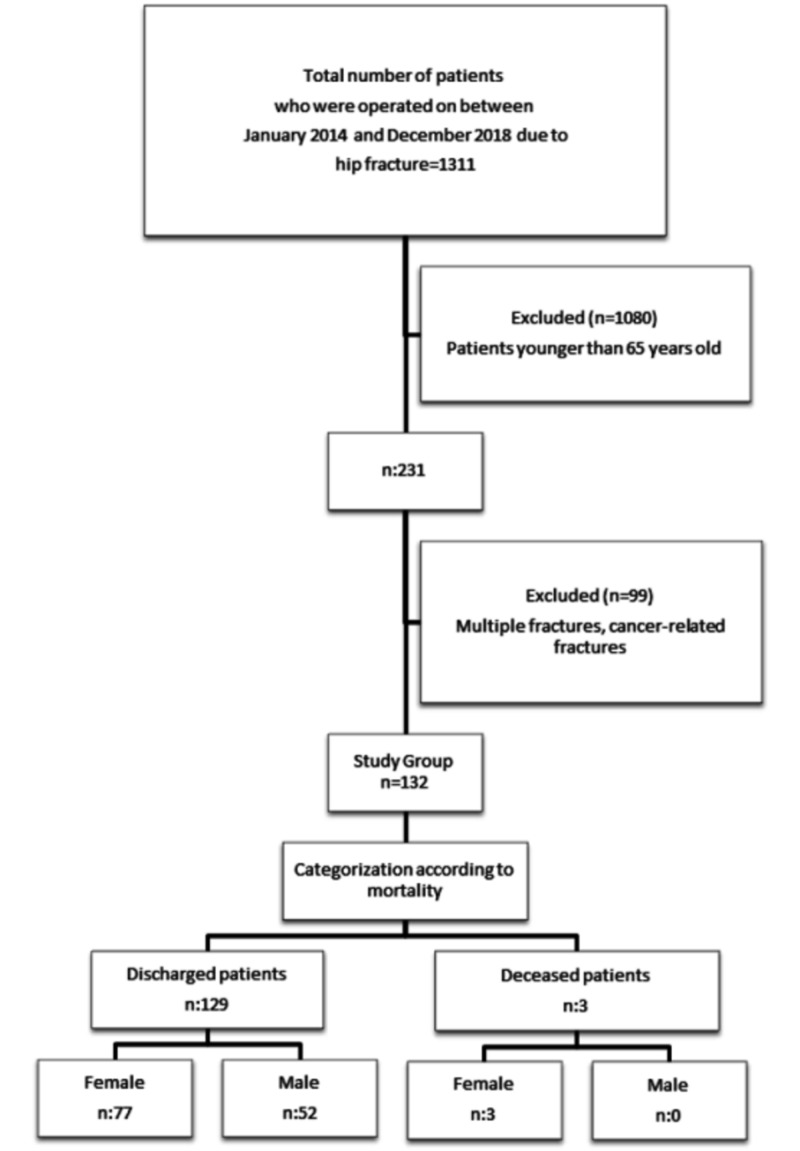
Diagram showing the study design and results

Duration of ICU stay showed positive moderate-level correlation with NLR 5 value (p < 0.001, r = 0.304) (Figure 1). Duration of ICU stay showed positive weak correlation with the magnitude of change between preoperative and postoperative NLR values (p = 0.021, r = 0.201) (Figure 2).

## Discussion

In our study, the increase in NLR measured at the time of admission and on the 5th day following surgery in patients aged over 65 years presenting to our clinic due to hip fracture was found to be closely associated with mortality.

Increased postoperative NLR following hip fracture has been reported as risk factor for postoperative mortality and cardiovascular complications. Forget et al. reported that in addition to advanced age, male gender, and comorbid diseases, a postoperative 5th day NLR value >5 following hip fracture surgery was associated with high postoperative mortality risk [3]. Temiz and Ersözlü stated that postoperative NLR ≥4.7 increased the risk of mortality in patients with hip fracture [5]. In our study, NLR was similar between discharged and deceased patients at the preoperative period. However, postoperative NLR was higher among deceased patients (p = 0.007). In addition, deceased patients showed greater change between preop and postop NLR values (p = 0.009). Preoperative NLR values were similar between patients admitted to the ICU and ward. Postoperative NLR was higher among patients who were admitted to the ICU (p = 0.007). Additionally, patients admitted to the ICU showed a greater increase rate of postoperative NLR values (p = 0.044).

In their studies, Jiang et al. and Soyalp et al. stated that advanced age was associated with higher risk of mortality [6-7]. Li et al. found increased anesthesia-related mortality in patients aged 75 years and above [8]. In our study, 72.8% (n = 94) of the discharged patients were older than 75 years. All of the deceased patients were aged 90 years or above (p = 0.005).

Several studies examining the waiting time before hip fracture operation reported that operations performed later than 24 hours or 48 hours resulted in increased mortality [9-10]. On the contrary, other studies reported that duration of preoperative waiting time did not change mortality rates [11-12]. In our study, 89.4% (n = 118) patients were operated on under elective settings while 10.6% (n = 14) of the patients had emergency operations. Of those who had emergency operations, 14.2% (n = 2) died in the ICU. The mortality rate was higher in patients who had emergency operations (p = 0.030).

In their study, Er et al. reported that of all cases treated with orthopedic and traumatologic interventions, 62.1% were male and 37.9% were female. In our study, the distribution of surgical cases shows predominance of females. Of all cases operated under elective settings, 61% were female and 39% were male, whereas of all cases operated on as emergency cases, 57.1% were female and 42.9% were male. No significant association was found between mortality and gender of operated cases (p = 0.278) [13].

In elderly patients, it is important to select the most appropriate surgical and anesthetic technique in order to reduce postoperative mortality. In their study, Topbas et al. reported that while general anesthesia was the method of choice for emergency operations, regional anesthesia was preferred for elective settings [14].

Although several studies reported that regional anesthesia was a safer option compared to general anesthesia for elderly patients, no significant difference has been documented so far between the two methods [15].

We examined the type of anesthesia administered to our patients, and accordingly, elective operations were performed under general anesthesia for 69.5% of patients and regional anesthesia for 30.5% of patients. As for emergency operations, 78.6% of patients were operated on under general anesthesia and 21.4% of patients under regional anesthesia. Of the deceased patients, two patients received general anesthesia and one patient received regional anesthesia. We did not find a significant association between the type of anesthesia and mortality (p = 0.711).

In our study, of all patients admitted to the ICU postoperatively, 71.3% received general anesthesia and 28.7% received regional anesthesia. There was no association between the type of anesthesia and postoperative ICU admission (p = 0.817).

Anemia is associated with higher prevalence of mortality, delirium, congestive heart failure, coronary artery disease, and falls in elderly patients. Several cohort studies have found anemia as an independent risk factor for mortality in elderly patients [16-17].

Zakai et al. reported that hemoglobin values below 12.7 mg/dl in women and 13.5 mg/dl in men were associated with increased mortality [18].

Gruson et al. found increased six month- and one year-mortality rates among anemic elderly patients who underwent hip fracture operation [19]. In our study, mean preoperative hemoglobin levels for women and men were 11.85 g/dl and 12.37 mg/dl, respectively. Mean preoperative hemoglobin levels were 12.2 g/dl for discharged patients and 12.6 g/dl for deceased patients. The association of hemoglobin level with mortality was not statistically significant (p = 0.982). Preoperative hemoglobin level showed a negative weak correlation with duration of hospital stay (p = 0.047, r = 0.173) and positive weak correlation with the amount of transfused erythrocyte units (p = 0.001, r = 0.282) (Table 2).

## Conclusions

Many factors can influence duration of hospital stay and mortality in patients with hip fracture. Determining new risk factors for mortality in elderly patients with hip fracture is very important. NLR is a simple and inexpensive marker and can be used as an objective risk factor for early postoperative mortality after hip fracture. We think that the increase in postoperative NLR in elderly patients who underwent surgery for hip fractures is important for predicting mortality and further research is needed.

## References

[1] Partridge JSL, Harari D, Martin FC, Dhesi JK (2014). The impact of pre-operative comprehensive geriatric assessment on postoperative outcomes in older patients undergoing scheduled surgery: a systematic review. Anaesthesia.

[2] Carrol K, Majeed A, Firth C, Gray J (2003). Prevalence and management of coronary heart disease in primary care: population-based cross-sectional study using a disease register. J Public Health Med.

[3] Forget P, Moreau N, Engel H, Cornu O, Boland B, De Kock M, Yombi JC (2015). The neutrophil-to-lymphocyte ratio (NLR) after surgery for hip fracture (HF). Arch Gerontol Geriatr.

[4] Cook EJ, Walsh SR, Farooq N, Alberts JC, Justin TA, Keeling NJ (2007). Post-operative neutrophil-lymphocyte ratio predicts complications following colorectal surgery. Int J Surg.

[5] Temiz A, Ersözlü S (2019). Admission neutrophil-to-lymphocyte ratio and postoperative mortality in elderly patients with hip fracture. Ulus Travma Acil Cerrahi Derg.

[6] Jiang HX, Majumdar SR, Dick DA, Moreau M, Raso J, Otto DD, Johnston DWC (2005). Development and initial validation of a risk score for predicting in-hospital and 1-year mortality in patients with hip fractures. J Bone Mineral Res.

[7] Soyalp C, Yuzkat N, Kılıc M, Akyol ME, Demir CY, Gulhas N (2019). Operative and prognostic parameters associated with elective versus emergency surgery in a retrospective cohort of elderly patients. Aging Clin Exp Res.

[8] Li G, Warner M, Lang BH, Huang L, Sun LS (2009). Epidemiology of anesthesia-related mortality in the United States, 1999-2005. Anesthesiology.

[9] Bottle A, Aylin P (2006). Mortality associated with delay in operation after hip fracture: observational study. BMJ.

[10] Moran CG, Wenn RT, Sikand M, Taylor AM (2005). Early mortality after hip fracture: is delay before surgery important?. J Bone Joint Surg Am.

[11] Grimes JP, Gregory PM, Noveck H, Butler MS, Carson JL (2002). The effects of time-to-surgery on mortality and morbidity in patients following hip fracture. Am J Med.

[12] Smektala R, Endres HG, Dasch B, Maier C, Trampisch HJ, Bonnaire F, Pientka L (2008). The effect of time-to-surgery on outcome in elderly patients with proximal femoral fractures. BMC Musculoskelet Disord.

[13] Er S, Çeğin MB, Göktaş U, Güner S, Yüzkat N (2015). Retrospective analysis of anesthesia methods in orthopaedics and traumatology surgery. (Article in Turkish). JARSS.

[14] Topbaş M, Çan G, Kızıl M, Yarış F (2002). Evaluation of the emergency and elective operations performed on elderly over 65 years in Karadeniz Technical University Medical School Farabi Hospital. (Article in Turkish). İnönü Üniversitesi Tıp Fakültesi Dergisi.

[15] Hepaguslar H, Elar Z (2003). Geriyatrik olgularda genel veya rejyonel anestezi seçimi. Türkiye Klinikleri J Anest Reanim.

[16] Chaves PH, Ashar B, Guralnik J, Fried LP (2002). Looking at the relationship between hemoglobin concentration and prevalent mobility difficulty in older women. Should the criteria currently used to define anemia in older people be reevaluated?. J Am Geriatr Soc.

[17] Ana BJ, Suman VJ, Fairbanks VF, Rademacher DM, Melton LJ (1997). Incidence of anemia in older people: an epidemiologic study in a well defined population. J Am Geriatr Soc.

[18] Zakai NA, Katz R, Hirsch C, Shlipak MG, Chaves PH, Newman AB, Cushman M (2005). A prospective study of anemia status, hemoglobin concentration, and mortality in an elderly cohort: the Cardiovascular Health Study. Arch Intern Med.

[19] Gruson KI, Aharonoff GB, Egol KA, Zuckerman JD, Koval KJ (2002). The relationship between admission hemoglobin level and outcome after hip fracture. J Orthop Trauma.

